# Haplotypes of TNF*α*/*β* Genes Associated with Sex-Specific Paranoid Schizophrenic Risk in Tunisian Population

**DOI:** 10.1155/2018/3502564

**Published:** 2018-12-05

**Authors:** Oumaima Inoubli, Achraf Jemli, Sihem Ben Fredj, Anouar Mechri, Lotfi Gaha, Besma Bel Hadj Jrad

**Affiliations:** ^1^Laboratory of Genetics, Biodiversity and Bioresource Valorization, Higher Institute of Biotechnology of Monastir, University of Monastir, Monastir, Tunisia; ^2^Department of Epidemiology, Farhat Hached University Hospital, Sousse, Tunisia; ^3^Department of Psychiatry and Vulnerability to Psychoses Laboratory-CHU Monastir, University of Monastir, Monastir, Tunisia

## Abstract

Several medical research findings have announced a strong association between the biology of cytokines and various brain activities. Since growing evidences suggest the crucial and complex role of the tumor necrosis factor in the CNS, we have hypothesized that functional genetic variants of the LTA and TNFA genes (LTA +252A/G (rs909253) and TNFA −857C/T (rs1799724) and TNFA −238G/A (rs361525)) may be involved in the predisposition to schizophrenia. This research is based on a case-control study. The RFLP-PCR genotyping was conducted on a Tunisian population composed of 208 patients and 208 controls. We found a strong significant overrepresentation of the minor alleles (G, T, and A, respectively) in all patients compared with controls (*p* = 0.003, OR = 1.55; *p* = 0.005, OR = 1.78; and *p* = 0.0001, OR = 1.74, respectively). This correlation was confirmed for male but not for female patients. Interestingly, the frequencies of the minor alleles were significantly more common among patients with paranoid schizophrenia when compared with controls (*p* = 0.003, OR = 1.75; *p* = 5 · 10^−6^, OR = 3.04; and *p* = 4 · 10^−6^, OR = 2.35, respectively). This potential association was confirmed by a logistic binary regression analysis only for the development of the paranoid form of schizophrenia (*p* = 0.001/OR = 2.6; *p* = 0.0002/OR = 3.2; and *p* = 0.0004/OR = 3.1, respectively) and remained not significant for the other subtypes. Moreover, our study showed an important association between GCA haplotype and the development of this pathological form (*p* = 10^−4^, OR = 3.71). In conclusion, our results proved a significant association between the three polymorphisms and paranoid schizophrenia, at least in the Tunisian population, suggesting a substantially increased risk for paranoid schizophrenia with dominant inheritance of these three minor alleles.

## 1. Introduction

An epidemiologic research has announced that 3.5% of the general population experience psychosis defined as serious mental disorders characterized by an impaired relationship with reality [1]. The prototypical psychotic disorder that affects more than 21 million people around the world (1% of the general population) is schizophrenia [1]. It is not only the most common psychosis but also schizophrenia tends to involve abnormalities in all of the symptom clusters (positive, negative, mood symptoms, and cognitive deficits) that tend to reliably develop during late adolescence and early adulthood (15–35 years) [2]. Several subtypes according to the distribution of symptoms as paranoid, undifferentiated, disorganized, and catatonic have been defined [3]. This disease was characterized with a lifetime morbidity risk of 0.5%–2.7% and heritability estimated at up to 80% [4]. Although multiple neurodevelopmental, neurotransmitter, neuroimmunology, and genetic hypothesis have been suggested to explain the aetiology of schizophrenia [5], it is not possible to claim that a single factor is responsible for the disorder.

Recent understanding suggests a significant role of chronic low-grade inflammation in the pathogenesis of schizophrenia based on its association with profound immune system disturbances both during prenatal development and in adult life [6, 7]. In fact, the direct evidence linking immune dysfunctions with this disease is represented by an increased level of proinflammatory cytokines in the peripheral nervous system as well as central nervous system (CNS) and microglial activation [6, 8, 9]. Importantly, IL-6 and TNF*α*, cytokines having novel roles in CNS development and functions including neuronal plasticity, cognition, and behavior, have been investigated quite extensively in schizophrenia [10]. TNF*α*, proinflammatory cytokine, exerts both homeostatic and pathophysiological roles in the central nervous system (CNS) [11]. In pathological conditions, astrocytes and mainly microglia release large amounts of TNF*α*. This de novo production of this cytokine is an important component of the so-called neuroinflammatory response that is associated with several neurological disorders [11–13]. Furthermore, disrupted TNF signaling is also found to cause altered development of the hippocampus and cognitive dysfunction [14, 15]. More than its stressor-like effects on CNS, including changes in tryptophan metabolism, hypothalamic-pituitary-adrenocortical axis, and brain-derived neurotrophic factor expression [16], this cytokine has been shown also to have a stimulatory effect on the catecholaminergic system; nevertheless, an inhibitory effect was detected with chronic TNF*α* release [17]. This is consistent with the hypothesis that the acute positive symptoms of schizophrenia are due to hyperdopaminergic activity, while the negative symptoms (which are more predominant in the chronic phase of schizophrenia) are the consequence of a relative hypodopaminergic activity [18]. All these evidences suggest that inflammatory hypothesis could be the main link between all the aetiological hypotheses of schizophrenia.

A postmortem study reported that inflammation-related genes are overexpressed in schizophrenia [19]. A 2014 genome-wide association study [20] identified 108 genetic loci (83 previously undetected) associated with schizophrenia and announced an important association with markers close to the major histocompatibility complex (MHC). The TNFA and LTA genes are located in tandem right within the class III region of MHC on chromosome 6 (6p21.1–6p21.3) [21]. TNF*α* (MIM 191160) and TNF*β* or lymphotoxin-alpha (LT-*α*; MIM 153440) are closely related cytokines that share 30% amino acid residues [22]. Several polymorphisms in the promoter region of TNF*α* and the intron 1 of TNF*β* have been associated with changes in the levels of circulating TNF*α* [23–28]. Since most of the previous studies have analyzed the TNFA −308G/A polymorphism association with schizophrenia, we choose in the present case-control study to investigate the impact of LTA +252A/G (rs909253), TNFA −857C/T (rs1799724), and TNFA −238G/A (rs361525) polymorphisms on the susceptibility to schizophrenia. Additionally, putting the accent on the heterogeneity of this disorder, we have examined differential distribution of genotypes and alleles in relation to sociodemographic and clinical characteristics of patients with schizophrenia. To our knowledge, this is the first study analyzing the combine association between these three single-nucleotide polymorphisms and the development of schizophrenia.

## 2. Materials and Methods

### 2.1. Case-Control Study Based on a Tunisian Middle Coast Population

#### 2.1.1. Patients

Schizophrenic patients (208) composed from 36 women (17%) and 172 men (83%); sex ratio = 4.77; they were diagnosed and treated in the Psychiatry Department of Fattouma Bourguiba University Hospital in Monastir. The permanent signs of the disturbance must persist for at least 6 months to consider the patient a true schizophrenic for our study. This period must include at least 1 month of active-phase symptoms. The actual age in this population varied from 19 to 66 years with a mean age = 39.06 ± 10.6 years, and the sociodemographic data including gender, birthplace, residence area, marital status, occupation, educational level, and socioeconomic level of each patient with the information about the duration of the illness, the scale of schizophrenia scores of positive (SAPS) and negative (SANS) syndromes, and the Brief Psychiatric Rating Scale (BPRS) were recorded.

The onset age was defined as the age of the emergence for the first positive psychotic symptoms, varied from 12 to 47 years with an average onset age in all patients of 24.9 ± 7.4 years. The diagnosis was focused on DSM IV axis I (*Diagnostic and Statistical Manual of Mental Disorders*) [3], with clinical subtype precision. Referring to the 4th revised edition of *Diagnostic and Statistical Manual of Mental Disorders* (DSM IV-TR) in 2000, the paranoid form of schizophrenia represents the most frequent subtype. In the present study, fifty-six (41.35%) patients met the criteria for undifferentiated, 86 (41.35%) for paranoid, and 36 (17.3%) for disorganized subtypes of schizophrenia.

The studied schizophrenic patients were hospitalized at least once, and they are in an acute psychotic episode. Only ongoing treatment was recorded. The patients are on first-generation antipsychotic treatments known as typical neuroleptic, at least on one generic with prolonged action (haloperidol (Haldol), fluphenazine (Modécate), or chlorpromazine (Largactil)) comedicated with atypical antipsychotics or antidepressants or anxiolytics or combined. The mean daily dosage was 385.2 ± 260.2 mg chlorpromazine equivalents. Additionally, more than half of the patients (56.7%) were on anticholinergic medication.

The selection was also based on the exclusion of schizoaffective disorder, mood disorder, and a general medical condition caused by a substance.

#### 2.1.2. Controls

This population contains blood donors with no personal or family history of psychiatric disorders recruited from Fattouma Bourguiba University Hospital in Monastir, Tunisia. A total of 208 controls divided into 159 men (76.4%) and 49 women (23.6%), with a sex ratio = 3.2, having an age varied from 18 to 69 years with a mean age = 32.7 ± 10.09 years, were included in the analysis for the three SNPs of the TNFA and LTA genes.

#### 2.1.3. Ethical Aspects

This study was approved by the institutional ethical committee of the Higher Institute of Biotechnology of Monastir, Tunisia. Its purpose and procedures were explained to all patients and controls, and a written informed consent was obtained from each patient and donor or from a family member.

#### 2.1.4. DNA Extraction

The extraction of the genomic DNA was carried from the venous blood sample on EDTA using the salting out method based on standard proteinase K digestion and salt purification. The first step consists on red blood cell lysis by a hemolytic solution (140 mM NH_4_Cl, 8.25 mM Tris, pH = 7). The pellet of white blood cells recovered after centrifugation was incubated overnight with proteinase K at 37°C and subsequently salted out at 4°C using a saturated NaCl solution. The DNA in the supernatant fluid was precipitated using concentrated solution of ethanol and dissolved in 400 *μ*L of ultrapure H_2_O.

#### 2.1.5. Polymorphism Analysis of the rs909253, rs1799724, and rs361525

The regions of interest were amplified by polymerase chain reaction according to Kim et al. [29] for +252A/G polymorphism using forward primer: 5′-CCG TGC TTC GTT TGG ACT A-3′ and reverse one: 5′-AGA GCT GGT GGG GAC ATG TCT G-3′. The genotyping of −857C/T and −238G/A polymorphisms was performed according to Skoog et al. [30] using forward primers: 5′-GGC TCT GAG GAA TGG GTT AC-3′ and 5′-AAA CAG ACC ACA GAC CTG GTC-3′, respectively, and reverse primers: 5′-CCT CTA CAT GGC CCT GTC TAC-3′ and 5′-CTC ACA CTC CCC ATC CTC CCG GAT C-3′, respectively.

The annealing temperature for each studied polymorphism was, respectively, 60°, 58°, and 59°. Polymerase chain reaction (PCR) products of the three sites +252A/G, −857C/T, and −238G/A were incubated with the appropriate restriction enzymes according to the manufacturer's instructions (NcoI, TaiI, and BamHI, respectively). The restriction fragments were separated by electrophoresis on 3% agarose gel stained with ethidium bromide.

We reanalyzed 30% of the samples randomly to validate the data generated by the PCR-RFLP method.

#### 2.1.6. Statistical Analysis

Allele frequencies of the three sites were tested by the Hardy–Weinberg equilibrium for both groups (patients and controls) using the chi-square test with one degree of freedom. The same test was used to evaluate the significant association between disease (schizophrenia against controls) and TNFA or LTA alleles or genotypes. Relative risks of schizophrenia associated with a particular genotype were estimated by the odds ratio (OR). The *χ*^2^ test was used to analyze the association of studied polymorphisms with the epidemiological and clinical parameters. All statistics were performed using the SPSS software (version 20, SPSS, Armonk, NY, USA). Logistic regression with categorical and quantitative independent variables was used, adjusting for the effects of age and gender through analysis of deviance from sequential addition of each variable. Haplotypes were analyzed using the SNPStats online software (https://www.snpstats.net/start.htm). Odds ratio with associated 95% confidence intervals (CI) was calculated. Haplotype frequencies are estimated using the implementation of the EM algorithm coded into the haplo.stats package. The Fisher exact test was performed to test the hypothesis of independence for the haplotypic distribution between cases and controls. The degree of linkage disequilibrium was determined by employing two standard measures, Lewontin's *D*′ [31] and the correlation coefficient *r*. Statistical significance of linkage disequilibrium was obtained through Monte Carlo permutation tests.

## 3. Results

Genotype distribution for both control and patient groups for all markers conformed to Hardy–Weinberg equilibrium. The distribution of genotype and allele frequencies of rs909253, rs1799724, and rs361525 in control subjects and patients with schizophrenia is shown in Table 1.

The frequency of the LTA +252 (GG) and TNFA −238 (AA) minor homozygote genotypes was higher among the patient group compared to the control ones (14.9% vs. 8.6% and 17.3% vs. 11.5%, respectively) with a significant difference (*p* = 0.01/OR = 2.26 and *p* = 0.002/OR = 2.5, respectively). Similarly, the minor allele frequencies of both LTA +252G and TNFA −238A were significantly higher in patients compared with the control group (37.3% vs. 27.6%; *p* = 0.003/OR = 1.55 and 43.75% vs. 30.8%; *p* = 0.0001/OR = 1.74, respectively). Differently, the frequency of TNFA −857 (TT) minor homozygote genotype was showed to be higher in patients than in control subjects but the difference did not reach statistical significance (3.3% vs. 1.5%; *p* = 0.1) potentially because of the low size of the population with this genotype. However, the allele frequency of TNF −857T was 16.3% in patients with schizophrenia and 9.8% in the control group, and the difference was statistically significant (*p* = 0.005/OR = 1.78). This result could be explained by the fact that the frequency of heterozygote genotype (CT) was significantly higher in the patient group than control ones (26% vs. 16.8%; *p* = 0.01/OR = 1.78).

These associations remain the most highly significant with the lowest Akaike information criterion (AIC) according to the dominant model of inheritance (Table 2).

As a research strategy, phenotypic markers can be incorporated into a genetic study to increase statistical power. Thus, we subdivided patients with schizophrenia into patients with and without specific characteristics to provide a robust reduction in the heterogeneity of this disorder. A gender stratification of the studied populations showed that the minor homozygote genotype as well as the minor allele frequencies of the three polymorphisms was strongly higher in male patients compared to male controls. Similar results have not been found for females in term of the three analyzed sites.

These results showed that the studied polymorphisms of LTA and TNFA genes remain associated with schizophrenia in male, not in female patients (see Table 1). Furthermore, when we stratified patients according to the clinical subtypes, the analysis showed a particular higher statistical difference between minor genotype and allele frequency distributions of the three gene sites in the paranoid form of the schizophrenic group than in the control ones. Result details are shown in Table 3.

As a genetic association study based on the exploration of a genetic factor involvement in the development of a multifactorial disease, a control of a possible bias of confounding factors which may interfere was mandatory. A logistic regression model including the variables of sex, age, and genotypes showed that the mutated genotypes LTA +252 (AG+GG), TNFA −857 (CT+TT), and TNFA −238 (GA+AA) remained risk factors only for the development of the paranoid form of schizophrenia (*p* = 0.001/OR = 2.6, *p* = 0.0002/OR = 3.2, and *p* = 0.0004/OR = 3.1, respectively). Thus, the association of the first intron of the LTA and the promoter regions of the TNFA polymorphisms seemed to be only specific for the paranoid subtype of schizophrenia and remained not significant for the other subtypes.

Moreover, the histogram analysis of the patient's onset age distribution showed two pics: the first one from 12 to 21 years (max value at 20 years) and the second one from 22 to 47 years (max value at 25 years). For this reason, we dichotomized the paranoid patients according to the onset age (less than 21 years and more than 22 years; see Table 4). Our results showed a significant association between minor alleles in the paranoid patients with both onset age groups, but this association was much more significant in the paranoid patients' group with an adult onset of schizophrenia compared to the control group according the three studied polymorphisms.

To evaluate the combine association effect of the three polymorphisms, we performed a haplotype analysis. Estimated haplotypes constructed for the three markers in both patients and controls are presented in Table 5. The results of this study pointed that 8 haplotypes are possible; all of them are expressed. The highest frequency of haplotypes in both control group and schizophrenic patients as well as according to the paranoid subtype was ACG (48.1%, 30.4%, and 22.4%, respectively). Statistically significant differences in the frequencies between cases and controls were observed for 4 out of the 8 haplotypes present (ACA, GCG, GCA, and ATA). However, five out of 8 haplotypes were found to be associated to the paranoid form (ACA, GCA, ATG, ATA, and GTA). The values of absolute *D*′ and *r* for controls are presented in Table 6. There was no evidence of linkage disequilibrium between markers, except for LTA +252A/G and TNFA −238G/A, where there was statistically significant evidence of linkage disequilibrium (*p* = 0.0001).

We found no significant association between the presence of a given polymorphism genotype and the other sociodemographic and clinical variables such as marital status, educational background, mean duration illness, occupational impairment, psychiatric hospitalization, symptomatology, and month of birth.

## 4. Discussion

Previous reports of genome-wide association studies have proved a significant association between schizophrenia and markers close to the major histocompatibility complex (MHC) region which contains gene coding for LTA and TNF*α*. Having noted that most of the previous studies analyzed the TNFA −308G/A polymorphism association with schizophrenia while the functional analysis of this SNP is divergent, we chose to examine other functional genetic polymorphisms within those genes' associations with schizophrenia in a Tunisian population.

To our knowledge, this is the first study investigating the combined impact of LTA and TNFA gene polymorphisms on susceptibility to schizophrenia. The present case-control study showed a strong significance association of the minor alleles +252G of LTA polymorphism and −857T and −238A of TNFA polymorphisms with the development of only paranoid schizophrenia subtype confirmed by the logistic regression model. This association was much more significant in the adult onset paranoid patients' group consistent with another study carried on TNFR2 polymorphism and the paranoid form in Tunisian population which is in favor for the chronic inflammation hypothesis implicated in the development of this disease [32].

Serum TNF*α* level does not change as long as symptoms of schizophrenia are present [33] indicating long periods of immune stimulation. Moreover, many evidences supported that TNFA/LTA-studied polymorphisms were involved in the modulation of gene expression both in vivo and in vitro in different stimulated cell types according to different conditions [23–28]. The results of such functional studies were very varying with negative and positive findings, which can be explained by the fact that age, body mass index, gender, time of the day, medicine intake, and several other factors could affect the TNF plasma levels [34]. Additionally, changes in behavior may probably be related to a local production of cytokines in the CNS and not through plasma levels [35]. Thus, it is not surprising that studies investigating the relationship between CNS disorders and cytokine plasma levels have inconsistent results.

As for TNF*β* which has an immunomodulator function [23, 30] and carries a predisposition to various immune diseases that frequently accompany mood disturbance [36, 37], TNFB∗G of the first intron TNFB +252A/G polymorphism seems also to be associated with high TNF*α* and TNF*β*production [23, 38]. A HaploChIP analyses demonstrate an altered polymerase II phosphorylation according to +252G>A polymorphism confirmed by RT-PCR suggesting that TNFB +252G is associated to an increased level of TNF*β* [39]. Furthermore, the expression of the TNFB mRNA in areas of white matter and gray matter in the brain has been revealed [40]. Another hypothesis suggests that TNF*α* expression is modulated through allele-specific binding of the transcription factor OCT-1 [41] and has been found to be associated with −857T in the promoter area, but not with −857C of the TNFA −857C/T polymorphism [42]. This T allele has been found to be associated with many inflammatory diseases, such as Crohn's disease, sarcoidosis, and rheumatoid arthritis in patients carrying the HLA-DR shared epitope [43–45]. For TNF −238 polymorphism, a putative repressor site is located between nucleotides −254 and −230, exactly where this site maps, and has been identified. This could push genetic transcription towards increased gene regulation on one hand due to a switch of guanine to adenine and on the other hand due to a better interaction of the guanine allele with a positive regulator of TNF production [46]. The TNF −238AA genotype and TNF −238A allele were found to be a marker of systemic lupus erythematosus and psoriasis [47, 48]. Moreover, analysis with the software TFSEARCH (http://www.cbrc.jp/research/db/TFSEARCH.html) predicted that the substitution of G to A at this position resulted in the abrogation of the binding of transcription factors such as ADR1 to this site.

Several evidences have announced an association between the increased level of this cytokine and various brain abnormalities. TNF*α* has been shown to induce cell death in septohippocampal cultures [49]. Increasingly, the presence of elevated TNF*α* concentrations leads to elevated glutamate concentration, thereby increasing the risk of glutamate neurotoxicity mediated by NMDA and not AMPA receptors in rat organotypic hippocampal slice cultures [50]. Furthermore, TNF*α* levels are elevated in several neuropathological states that are associated with learning and memory deficits, leading to the search for a possible role in plasticity. To this end, works carried out in the hippocampus demonstrate that TNF*α* regulate the development of the hippocampus, as TNF-R1 and TNF-R2 knockout mice demonstrate decreased arborization of the apical dendrites of the CA1 and CA3 regions and accelerated dentate gyrus development [51], and altered regions in schizophrenia as well.

A few reports are published on the association between schizophrenia and the present genetic polymorphisms of LTA and TNFA genes. A study carried on the Korean population suggested that the TNFB polymorphism may confer susceptibility to schizophrenia [52]. In another study on the Saudi population [53], LTA (+252A/G) polymorphism showed to be not directly associated with schizophrenia. For the −857C>T polymorphism, two research teams announced no association with schizophrenia in Chinese populations [54, 55]. No significant differences in allele frequencies were observed for TNFA −238 variant between the patient and control groups in Indian Bengali population [56], in Chinese Han population [54], and in Australian, Indian Fijian, Indigenous Fijian, and Brahmin populations [57]. Different results are likely explained by ethnicity differences which have been reported to be strongly associated with cytokine gene polymorphisms. In fact, the TNF genes are situated within the MHC region, which is known to be both highly polymorphic and subjected to ethnic variation [58]. In contrast, the analyzed SNPs in our work have been tightly associated with several pathologies with chronic inflammation such as type 1 diabetes, multiple sclerosis, and cancer [59–61].

The other interesting result of the present study was gender-specific association of all the analyzed polymorphisms. The minor allele of the three sites was significantly more frequent in male patients than in healthy males, whereas this association was not shown in females. Indeed, the allele frequency distributions have been carried out in a larger cohort of female controls (*n* = 80) vs. the studied female patients (*n* = 36) not only to verify the present distributions but also to strengthen the power of this statistical correlation. The result of genotyping 80 healthy females showed no differences with the announced distributions (TNFB +252A 70%/G 30%, TNFA −857C 83.7%/T 16.3%, and TNFA −238G 64.4%/A 35.6%), and the correlation remained statistically insignificant (*p* = 0.9, OR = 1.02 (0.55–1.87); *p* = 0.5, OR = 1.2 (0.59–2.5); and *p* = 0.6, OR = 1.1 (0.64–2.04), respectively). The additional female controls were not considered in the present research work since the sex ratio represents a confounding factor in a genetic association study. In fact, on one hand, the two populations (case-control) have to be matched in the sex ratio and age to maximize the power of adjusted statistical results by logistic regression. On the other hand, the incidence of schizophrenia has been marked by gender differences. Using standard diagnostic criteria in an incidence population study, a meta-analysis by Aleman et al. confirmed that men had a higher incidence than women [62]. In Tunisia, a study carried on hospitalized schizophrenic patients in the biggest hospital of mental diseases in the country has announced that female patients with schizophrenia are three times less to be hospitalized than men (during 2003; 174 men vs. 54 women), same incidence in the hospital where the sample are collected [63].

Nevertheless, the hypothesis suggesting that a larger female cohort would possibly yield a significant correlation remains valid. Thus, it is recommended to prove this nonassociation with female gender not only in the Tunisian middle coast population but also in a larger Tunisian cohort (female controls vs. female patients).

This higher frequency of the different alleles in male patients, which is known to be associated with increased production of TNF*α*, might be responsible for the higher prevalence of schizophrenia in male subjects than in female subjects. The interaction between the TNF level and sex was also observed in a variety of normal physiologic states and complex diseases characterized by chronic inflammation [64–66]. These differences include the possible influence of sex hormones, potentially through the shift from TH1 to TH2 cell response [67]. In fact, during the luteal phase of the ovarian cycle, when the estrogen level is high, the immune response is shifted towards a TH2-type response, as reflected by increased IL-4 production in this phase of the cycle [68]. In addition, an estrogen inhibitory element was mapped to the TNF*α* (TH1 cytokine) promoter [69], which might explain the inhibitory effect of estrogens on the expression of TNF*α* in target cells [70]. More interestingly, ethinyl estradiol, like 17*β*-estradiol, drastically suppressed EAE which is an inflammatory demyelinating disease of the CNS and significantly decreased the secretion of proinflammatory cytokines (IFN-*γ*, TNF*α*, and IL-6) by activated T cells but increased the expression of TGF-*β*3 in the CNS [71]. These results may too explain the early onset age of schizophrenia in male subjects.

Given that the previous study showed distinct molecular phenotypes in male and female schizophrenia patients [72–75], our present data provided additional evidence supporting the role of gender differences in the aetiology of schizophrenia and could represent a proof of the phenotype “dissection” strategy to strengthen the informative power of genetic association studies.

For a better understanding and valorization of this SNP association, we carried out a haplotype analysis to determine whether combinations of specific alleles are associated with higher risk of developing schizophrenia. It was found that four haplotypes are correlated with increasing risk of schizophrenia development in Tunisian population and five of them are associated to the paranoid subtype. In fact, these results showed three shared haplotypes which are associated to the hall studied schizophrenic population and more specifically to the paranoid subtype (ACA, GCA, and ATA). The analysis has demonstrated a linkage disequilibrium between +252A>G LTA and −238G>A TNFA, result explaining the high significance association of haplotypes containing at least one of the minor alleles +252G or −238A or both. Most interestingly, the haplotype combining these two minor alleles GCA was more expressed in paranoid patients compared to the control group with the highest significant difference (*p* = 0.0001, OR = 3.71). Furthermore, the haplotypes combining these two alleles were found to be associated to the development of other inflammatory diseases [76, 77]. A study on the Tunisian population investigating the association between TNFA promotor polymorphisms and type 1 diabetes showed that the haplotypes containing the TNFA −857C and TNFA −238G are the most likely haplotypes associated to the development of this disease [61] which is consistent with our result showing the association between GCG haplotype and global schizophrenia. Our present study can be considered the first study investigating this correlation with schizophrenia.

In conclusion, at least in the Tunisian middle coast population, the promoter regions of the TNFA and the first intron polymorphism of the LTA or the other genes at proximity substantially seem to be implicated in the development of schizophrenia, specifically of the paranoid subtype. The presence of the minor alleles increases the risk of developing paranoid schizophrenia. Further studies are needed to fine map this gene-rich MHC region to unravel the role of the causative gene variant in the immunogenetics of schizophrenia.

## Figures and Tables

**Table 1:** Distribution of genotype and allele frequencies of rs909253, rs1799724, and rs361525 in control subjects and patients with schizophrenia.

	Controls (*n* = 208)	Patients (*n* = 208)	*p* value	OR (95% CI)	Controls	Patients	*p* value	OR (95% CI)	Controls	Patients	*p* value	OR (95% CI)
					Males (*n* = 159)	Males (*n* = 172)			Females (*n* = 49)	Females (*n* = 36)		
*rs909253*												
AA	111 (53.4)	84 (40.4)	1		85 (53.4)	67 (38.9)	1		26 (53.1)	17 (47.2)	1	
AG	79 (38)	93 (44.7)	0.03^∗^	1.55 (1.02–2.35)	61 (38.4)	77 (44.8)	0.04^∗^	1.59 (1.005–2.55)	18 (36.7)	16 (44.5)	0.5	1.35 (0.53–3.41)
GG	18 (8.6)	31 (14.9)	0.01^∗^	2.26 (1.19–4.40)	13 (8.2)	28 (16.3)	0.006^∗^	2.71 (1.31–5.80)	5 (10.2)	3 (8.3)	0.9	0.91 (0.16–4.51)
*Allele*												
A	301 (72.4)	261 (62.7)	1		231 (72.6)	211 (61.3)	1		70 (71.4)	50 (69.4)	1	
G	115 (27.6)	155 (37.3)	0.003^∗^	1.55 (1.16–2.08)	87 (27.4)	133 (38.7)	0.002^∗^	1.67 (1.20–2.32)	28 (28.6)	22 (30.6)	0.7	1.09 (0.55–2.14)

*rs1799724*												
CC	170 (81.7)	147 (70.7)	1		133 (83.7)	124 (72.1)	1		37 (75.5)	23 (63.9)	1	
CT	35 (16.8)	54 (26)	0.01^∗^	1.78 (1.1–2.89)	25 (15.7)	42 (24.4)	0.03^∗^	1.79 (1.03–3.15)	10 (20.4)	12 (33.3)	0.2	1.91 (0.7–5.29)
TT	3 (1.5)	7 (3.3)	0.1	2.69 (0.69–13.01)	1 (0.6)	6 (3.5)	0.06	6.39 (0.92–150.1)	2 (4.1)	1 (2.8)	0.9	0.8 (0.02–11.12)
*Allele*												
C	375 (90.2)	348 (83.7)	1		291 (91.5)	290 (84.3)	1		84 (85.7)	58 (80.6)	1	
T	41 (9.8)	68 (16.3)	0.005^∗^	1.78 (1.18–2.71)	27 (8.5)	54 (15.7)	0.004^∗^	2.005 (1.23–3.31)	14 (14.3)	14 (19.4)	0.3	1.44 (0.63–3.3)

*rs361525*	*n* (%)	*n* (%)			*n* (%)	*n* (%)			*n* (%)	*n* (%)		
GG	104 (50)	62 (29.8)	1		83 (51.2)	48 (27.9)	1		21 (42.9)	14 (38.9)	1	
GA	80 (38.5)	110 (52.9)	0.0001^∗^	2.30 (1.50–3.53)	57 (35.9)	94 (54.7)	0.00001^∗^	2.84 (1.75–4.63)	23 (46.9)	16 (44.4)	0.9	1.04 (0.40–2.68)
AA	24 (11.5)	36 (17.3)	0.002^∗^	2.50 (1.37–4.63)	19 (11.9)	30 (17.4)	0.003^∗^	2.71 (1.38–5.41)	5 (10.2)	6 (16.7)	0.4	1.77 (0.43–7.50)
*Allele*												
G	288 (69.2)	234 (56.25)	1		223 (70.1)	187 (54.4)	1		65 (66.3)	44 (61.1)	1	
A	128 (30.8)	182 (43.75)	0.0001^∗^	1.74 (1.31–2.32)	95 (29.9)	157 (45.6)	0.00002^∗^	1.96 (1.43–2.71)	33 (33.7)	28 (38.9)	0.4	1.25 (0.66–2.36)

^∗^ *p* < 0.05. The chi-square test was used to evaluate the significant association between disease (schizophrenia against controls) and TNFA or LTA alleles or genotypes. The Fisher exact test was used to determine whether significant differences were observed when the patient group was compared with the control group and having total less than 5.

**Table 2:** Distribution of genotype frequencies of rs909253, rs1799724, and rs361525 between controls and patients according to the different models of inheritance.

	Controls *n* (%)	Patients *n* (%)	*p* value	OR (95% CI)	AIC
rs909253					
*Codominant*					
AA	111 (53.4)	84 (40.4)	1		
AG	79 (38)	93 (44.7)	0.03^∗^	1.55 (1.02–2.35)	
GG	18 (8.6)	31 (14.9)	0.01^∗^	2.26 (1.19–4.40)	574.3
*Dominant*					
AA	111 (53.4)	84 (40.4)	1		
AG+GG	97 (46.6)	124 (59.6)	0.008^∗^	1.68 (1.14–2.49)	573.6
*Recessive*					
AA+AG	190 (91.4)	177 (85.1)	1		
GG	18 (8.6)	31 (14.9)	0.049^∗^	1.84 (1.001–3.47)	576.7

rs1799724					
*Codominant*					
CC	170 (81.7)	147 (70.7)	1		
CT	35 (16.8)	54 (26)	0.01^∗^	1.78 (1.1–2.89)	
TT	3 (1.5)	7 (3.3)	0.1	2.69 (0.69–13.01)	575.3
*Dominant*					
CC	170 (81.7)	147 (70.7)	1		
CC+CT	38 (18.3)	61 (29.3)	0.008^∗^	1.85 (1.17–2.95)	573.6
*Recessive*					
CC+CT	205 (98.5)	201 (96.6)	1		
TT	3 (1.5)	7 (3.4)	0.2	2.37 (0.61–11.44)	579

rs361525					
*Codominant*					
GG	104 (50)	62 (29.8)	1		
GA	80 (38.5)	110 (52.9)	0.0001^∗^	2.30 (1.50–3.53)	
AA	24 (11.5)	36 (17.3)	0.002^∗^	2.50 (1.37–4.63)	564.9
*Dominant*					
GG	104 (50)	62 (29.8)	1		
GA+AA	104 (50)	146 (70.2)	0.00002^∗^	2.35 (1.57–3.52)	562.9
*Recessive*					
GG+GA	184 (88.5)	172 (82.7)	1		
AA	24 (11.5)	36 (17.3)	0.09	0.62 (0.35–1.08)	577.9

^∗^ *p* < 0.05. The chi-square test was used to evaluate the significant association between disease (schizophrenia against controls) and TNFA or LTA alleles or genotypes. AIC (Akaike information criterion) provides a means for model selection.

**Table 3:** Distribution of genotype and allele frequencies of rs909253, rs1799724, and rs361525 in control subjects and patients with different subtypes of schizophrenia.

	Genotype	*n* (%)		*p* value	OR (95% CI)	Allele	*n* (%)	*p* value	OR (95% CI)
*rs909253*	AA	AG	GG			A	G		
Controls (*n* = 208)	111 (53.4)	79 (38)	18 (8.6)			301 (72.4)	115 (27.6)		
Undifferentiated type (*n* = 86)	36 (41.9)	40 (46.5)	10 (11.6)	0.07	1.58 (0.95–2.65)	112 (65.1)	60 (34.9)	0.08	1.4 (0.95–2.04)
Disorganized type (*n* = 36)	15 (41.7)	16 (44.4)	5 (13.9)	0.2	1.59 (0.77–3.33)	46 (63.9)	26 (36.1)	0.1	1.47 (0.86–2.49)
Paranoid type (*n* = 86)	33 (38.4)	37 (43)	16 (18.6)	0.01^∗^	1.83 (1.1–3.08)	103 (59.9)	69 (40.1)	0.003^∗^	1.75 (1.20–2.54)

*rs1799724*	CC	CT	TT			C	T		
Controls (*n* = 208)	170 (81.7)	35 (16.8)	3 (1.5)			375 (90.2)	41 (9.8)		
Undifferentiated type (*n* = 86)	72 (83.7)	13 (15.1)	1 (1.2)	0.6	0.87 (0.4–1.68)	157 (91.3)	15 (8.7)	0.6	0.87 (0.45–1.60)
Disorganized type (*n* = 36)	28 (77.8)	6 (16.7)	2 (5.5)	0.5	1.27 (0.5–2.9)	62 (86.1)	10 (13.9)	0.3	1.47 (0.67–3.03)
Paranoid type (*n* = 86)	47 (54.7)	35 (40.7)	4 (4.6)	3 · 10^−6∗^	3.69 (2.12–6.44)	129 (75)	43 (25)	5 · 10^−6∗^	3.04 (1.89–4.89)

*rs361525*	GG	GA	AA			G	A		
Controls (*n* = 208)	104 (50)	80 (38.5)	24 (11.5)			288 (69.2)	128 (30.8)		
Undifferentiated type (*n* = 86)	31 (36.05)	43 (50)	12 (13.95)	0.02^∗^	1.77 (1.05–2.99)	105 (61.05)	67 (38.95)	0.05	1.43 (0.98–2.07)
Disorganized type (*n* = 36)	14 (38.9)	17 (47.2)	5 (13.9)	0.2	1.56 (0.76–3.30)	45 (62.5)	27 (37.5)	0.2	1.34 (0.79–2.26)
Paranoid type (*n* = 86)	17 (19.8)	50 (58.1)	19 (22.1)	9 · 10^−7∗^	4.04 (2.25–7.50)	84 (48.8)	88 (51.2)	4 · 10^−6∗^	2.35 (1.63–3.39)

This represented association was observed under the dominant model. ^∗^*p* < 0.05. The chi-square test was used to evaluate the significant association between disease (schizophrenia against controls) and TNFA or LTA alleles or genotypes. The Fisher exact test was used to determine whether significant differences were observed when the patient group was compared with the control group and having total less than 5.

**Table 4:** Distribution of genotype and allele frequencies of rs909253, rs1799724, and rs361525 in control subjects and paranoid-type patients according to onset age.

	Genotype (%)		*p* value (OR)	Allele (%)		*p* value (OR)
*rs909253*	AA	AG+GG		A	G	
Controls	111 (53.4)	97 (46.6)		301 (72.4)	115 (27.6)	
Paranoid type ≤ 21 yrs (*n* = 23)	9 (39.1)	14 (60.9)	0.2 (1.77)	26 (56.5)	20 (43.5)	0.03 (2.01)^∗^
Paranoid type > 21 yrs (*n* = 59)	23 (39)	36 (61)	0.05 (1.78)^∗^	73 (61.9)	45 (38.1)	0.03 (1.6)^∗^

*rs1799724*	CC	CT+TT		C	T	
Controls	170 (81.7)	38 (18.3)		375 (90.2)	41 (9.8)	
Paranoid type ≤ 21 yrs (*n* = 23)	13 (56.5)	10 (43.5)	0.01 (3.41)^∗^	34 (73.9)	12 (26.1)	0.003 (3.2)^∗^
Paranoid type > 21 yrs (*n* = 59)	32 (54.2)	27 (45.8)	3 · 10^−5^ (3.75)^∗∗^	89 (75.4)	29 (24.6)	9 · 10^−5^ (2.9)^∗∗^

*rs361525*	GG	GA+AA		G	A	
Controls	104 (50)	104 (50)		288 (69.2)	128 (30.8)	
Paranoid type ≤ 21 yrs (*n* = 23)	5 (21.7)	18 (78.3)	0.01 (3.58)^∗^	24 (52.2)	22 (47.8)	0.02 (2.05)^∗^
Paranoid type > 21 yrs (*n* = 59)	12 (20.3)	47 (79.7)	3 · 10^−5^ (3.89)^∗∗^	57 (48.3)	61 (51.7)	3 · 10^−5^ (2.4)^∗∗^

^∗^ *p* < 0.05. ^∗∗^ highly significant.

**Table 5:** TNF*α* haplotype frequencies of the rs909253, rs1799724, and rs361525 in control subjects, patients with schizophrenia, and according to paranoid subtype.

Haplotype	rs909253	rs1799724	rs361525	Controls % (*N* = 208)	Patients % (*N* = 208)	OR (95% CI)	*p* value
1	A	C	G	48.1	30.4	1	
2	A	C	A	16.7	21.4	2.07 (1.27–3.41)	0.003^∗^
3	G	C	G	14.4	17.8	2.03 (1.21–3.41)	0.007^∗^
4	G	C	A	11	14.1	1.98 (1.20–3.25)	0.007^∗^
5	A	T	G	5.2	5.5	1.87 (0.73–4.80)	0.19
6	A	T	A	2.4	5.5	3.45 (1.28–9.30)	0.015^∗^
7	G	T	G	1.5	2.5	1.73 (0.41–7.21)	0.45
8	G	T	A	0.7	2.8	7.99 (0.95–67.00)	0.056

Haplotype	rs909253	rs1799724	rs361525	Controls %	Paranoid%	OR (95% CI)	*p* value
1	A	C	G	48.1	22.4	1	
2	A	C	A	16.7	21.2	2.87 (1.35–6.12)	0.006^∗^
3	G	C	G	14.4	12	2.07 (0.85–5.06)	0.11
4	G	C	A	11	19.1	3.71 (1.91–7.21)	0.0001^∗^
5	A	T	G	5.2	9.7	6.20 (1.74–22.08)	0.005^∗^
6	A	T	A	2.4	6.7	6.14 (1.94–19.45)	0.002^∗^
7	G	T	G	1.5	4.5	3.23 (0.58–18.06)	0.18
8	G	T	A	0.7	4.1	22.75 (1.39–371.31)	0.029^∗^

^∗^ *p* < 0.05.

**Table 6:** Allele frequencies and pairwise linkage disequilibrium coefficients of the three analyzed polymorphisms in TNFA and LTA genes in 208 Tunisian control subjects.

SNP	Allele frequencies	Absolute *D*′ (Pearson's *r*)		*p* value	
		TNFA −238(G/A)	TNFA −857(C/T)	TNFA −238(G/A)	TNFA −857(C/T)
LTA +252(A/G)	0.72/0.28	0.128 (0.115)	0.078 (−0.021)	0.0001^∗^	0.54
TNFA −857(C/T)	0.9/0.1	0.103 (0.051)	—	0.13	—
TNFA −238(G/A)	0.69/0.31	—	—	—	—

^∗^ *p* < 0.05.

## Data Availability

No dataset were used to support this study.
